# Understanding symptom clusters, diagnosis and healthcare experiences in myalgic encephalomyelitis/chronic fatigue syndrome and long COVID: a cross-sectional survey in the UK

**DOI:** 10.1136/bmjopen-2024-094658

**Published:** 2025-04-02

**Authors:** Maedeh Mansoubi, Thomas Richards, Martine Ainsworth-Wells, Russell Fleming, Phaedra Leveridge, Charles Shepherd, Helen Dawes

**Affiliations:** 1 Medical School, University of Exeter, Exeter, UK; 2 NIHR, BioMedical Research Centre (BRC), Medical School, University of Exeter, Exeter, UK; 3 ME Association, London, UK; 4 ME Association, Buckingham, UK

**Keywords:** COVID-19, Primary Care, Fatigue, Self-Management

## Abstract

**Abstract:**

**Objectives:**

This study aims to provide an in-depth analysis of the symptoms, coexisting conditions and service utilisation among people with myalgic encephalomyelitis/chronic fatigue syndrome (ME/CFS) and long COVID. The major research questions include the clustering of symptoms, the relationship between key factors and diagnosis time, and the perceived impact of National Institute for Health and Care Excellence (NICE) guidelines on patient care.

**Design:**

Cross-sectional survey using secondary data analysis.

**Setting:**

Community-based primary care level across the UK, incorporating online survey participation.

**Participants:**

A total of 10 458 individuals responded to the survey, of which 8804 confirmed that they or a close friend/family member had ME/CFS or long COVID. The majority of respondents were female (83.4%), with participants from diverse regions of the UK.

**Primary and secondary outcome measures:**

Primary outcomes included prevalence and clustering of symptoms, time to diagnosis, and participant satisfaction with National Health Service (NHS) care, while secondary outcomes focused on symptom management strategies and the perceived effect of NICE guidelines.

**Results:**

Fatigue (88.2%), postexertional malaise (78.2%), cognitive dysfunction (88.4%), pain (87.6%) and sleep disturbances (88.2%) were the most commonly reported symptoms among participants with ME/CFS, with similar patterns observed in long COVID. Time to diagnosis for ME/CFS ranged widely, with 22.1% diagnosed within 1–2 years of symptom onset and 12.9% taking more than 10 years. Despite updated NICE guidelines, only 10.1% of participants reported a positive impact on care, and satisfaction with NHS services remained low (6.9% for ME/CFS and 14.4% for long COVID).

**Conclusions:**

ME/CFS and long COVID share overlapping but distinct symptom clusters, indicating common challenges in management. The findings highlight significant delays in diagnosis and low satisfaction with specialist services, suggesting a need for improved self-management resources and better-coordinated care across the NHS.

**Strengths and limitations of this study**

- The large sample size (n=10 458) strengthens the reliability and validity of the results.
- This project has evaluated services and management strategies for both myalgic encephalomyelitis and long COVID conditions.
- The online survey format facilitated participation from a geographically diverse population across the UK, ensuring broad representation.
- Reliance on self-reported diagnoses may have introduced potential bias and variability in diagnostic accuracy.

## Introduction

 ME/CFS (myalgic encephalomyelitis/chronic fatigue syndrome) is a serious, complex and systemic disease associated with neurological, immunological, autonomic and energy metabolism dysfunction.^1^ The WHO classified it in ICD-10 G93.3 as a disease of the central nervous system.^2^ Diagnostic criteria for ME/CFS in the 2021 National Institute for Health and Care Excellence (NICE) guideline state that four key symptoms must be present: debilitating fatigue that is made worse by activity, cognitive dysfunction (brain fog), unrefreshing sleep and sleep disturbance, and postexertional malaise (PEM).^3^ At present, there is no effective treatment for ME/CFS, partly due to a lack of understanding of the underlying disease process. Therefore, management aims to reduce symptoms where possible and to pace physical and mental activities to avoid PEM.^3^

Similarly, after acute COVID-19, approximately 10% of patients remain unwell for months with persistent fatigue, cognitive problems, headaches, disrupted sleep, myalgias and arthralgias, PEM, orthostatic intolerance, and other symptoms affecting functioning, with those more severely affected being housebound and disabled.^4^ Long COVID (also known as post-COVID-19 condition) is a heterogeneous illness that follows acute infection with the SARS-CoV-2 virus, which persists for 3 months or more after a COVID-19 infection. Common symptoms of long COVID include fatigue, cognitive dysfunction and breathlessness, with a wide range of other symptoms that may predominate in some patients.^5 6^ Evidence exists for multiple pathophysiological mechanisms in long COVID.^7 8^ As such, long COVID has similarities with ME/CFS.^9^ The similarities and differences in presentation and biology have recently been described in a recent review, highlighting abnormalities of the central and autonomic nervous system, lungs, heart, vasculature, immune system, gut microbiome, energy metabolism and redox balance.^8^ Considering the impact and similarity of these diseases, the need to highlight challenges within both conditions and set out priorities for future investigation and clinical management has been emphasised.^8^

The 2021 ME/CFS NICE guideline provides key recommendations that consider current evidence. These guidelines include principles of care for people with ME/CFS, suspecting ME/CFS, advice for people with suspected ME/CFS, diagnosis, assessment, and care and support planning by an ME/CFS specialist team, information and support, safeguarding, access to care and support, supporting people with ME/CFS in work, education, and training, multidisciplinary care, managing ME/CFS, symptom management for people with ME/CFS, managing coexisting conditions, managing flare-ups in symptoms and relapse, review in primary care, training for health and social care professionals, and care for people with severe or very severe ME/CFS.^3^ The guidelines highlight the absence of evidence supporting optimal care and management. Similarly, in 2021, new NICE guidelines for COVID-19 were also developed, and an overview of the management of this condition was presented,^9^ although the guidance for long COVID is less explicitly stated.

Here, we set out to describe the views and opinions of people living with ME/CFS and long COVID within the same survey. Whereas the guidelines for children and young people (CYP) with ME/CFS have been evaluated, to date, there has been no evaluation for adults. The evaluation of 92 CYPs highlighted key areas for improvement, including lack of investigation, referral and poor management, particularly for CYPs with severe ME/CFS who could not leave their home.^10^ We report on the views of people living with ME/CFS and those in people with long COVID in areas associated with the key recent NICE recommendations, aiming to target key areas for both future research and current clinical management.

## Methods

### Aims and objectives

To describe current practices and experiences of people with ME/CFS and with long COVID mapped to key NICE guidelines.

### Objectives

1. To describe in long COVID and ME/CFS participants, with and without a diagnosis, key demographics, diagnosis time, symptoms, comorbidities and treatments, experiences around support from the National Health Service (NHS) and social care, and key symptom management strategies.
2. To describe symptoms and clustering of symptoms for long COVID and ME/CFS.
3. To describe the extent of the relationship of key factors relating to time to diagnosis.
4. To describe the time to diagnosis in relation to the year diagnosed.
5. To determine the impact of the NICE guideline on ME/CFS on patients’ lives and the service they received since its publication in October 2021.

### Design

This is a cross-sectional survey of the current practices and experiences of people with ME/CFS and long COVID.

This study involved a secondary analysis of anonymised survey data collected to explore the experiences of individuals with ME/CFS and long COVID. The data were analysed in a secure environment in accordance with the University of Exeter’s data protection policies. No new data were collected, and the secondary analysis was conducted within the scope of the original data collection purpose, as outlined to participants at the time of the survey.

### Survey methods

An online survey was open for participation between 22 May and 31 July 2023. Eligibility was self-assessed, and the survey was available in English. People with suspected or a diagnosis of ME/CFS or long COVID were invited to participate in answering questions to understand their experiences with diagnosis, symptom management and NHS care, as well as map out coverage and care for people in the UK and determine areas for improvement. A blank copy of the survey measure has been included as a online supplemental file 1. Consent for secondary anonymous data analysis was carried out as follows. The opening page of the survey served as the participant information sheet and consent form for data to be analysed independently. Secondary data analysis was performed on the anonymous data by the University of Exeter following confirmation from the University sponsor (2023).

No formal a priori sample size calculation was performed, and the number of responses during the study period determined the sample size. However, it was estimated that approximately 1000 people would be recruited. Considering an estimated prevalence of 250–265 000 in the UK and a confidence level of 95%, 664 people would give a margin of error of 5%.

A self-selected sample of people on the ME Association https://meassociation.org.uk mailing lists and those that clicked through digital ads on relevant websites. The study aims of the project and how the results would be used were clearly stated to respondents to inform their choice to participate as follows: (a) inform more people about ME/CFS and long COVID to help them obtain an accurate diagnosis if needed and find the right support to improve their life quality; (b) produce an independent report that will raise awareness and help improve the standard of healthcare by working with the NHS and social care services. Participants could only take the survey once but were allowed to complete it in multiple sessions to accommodate fatigue for up to 3 days if they were too fatigued to complete it in one go. Participants had to confirm whether they were completing for themselves or for a close friend/family member had a diagnosis or symptoms of ME/CFS or long COVID. For respondents without a formal diagnosis, the survey asked them to confirm the presence of four key symptoms (fatigue, PEM, cognitive dysfunction and unrefreshing sleep) and their persistence for at least 3 months, as outlined in the NICE guidelines. For those reporting a current diagnosis, no specific question was included to confirm adherence to NICE or other diagnostic criteria.

### Variables

Data were collected on demographics, diagnosis, symptoms, comorbidities and treatments, experiences around NHS and social care support, and key symptom management strategies.

#### Data sources

All data were obtained via an online survey using Qualtrics. The MEA Association and One Minute to Midnight designed the survey in consultation with people with ME/CFS, who trialled the survey before being released. Considering feedback to minimise burden and optimise ease of use, the questionnaire was bifurcated so that participants did not progress to questions that were not relevant to them (ie, those with long COVID were not asked ME/CFS questions). Participants accessed the survey via a device with an internet connection (phone, computer and tablet). Names or other identifying details were not collected.

### Missing data

The current study has not imputed missing data, and the number of responses at variable levels is reported in the results/Appendix. Missing data were handled by incorporating all available responses for each analysis. Participants were not excluded due to incomplete survey submissions, and analyses were conducted using the maximum number of responses available for each variable. The number of respondents included in each analysis is transparently reported in the Results section.

### Statistics

Descriptive analyses were conducted to characterise gender, age, diagnoses, symptoms, comorbidities, treatments, experiences of support from NHS and social care, and key symptom management strategies. These were reported as frequencies and percentages for both ME/CFS and long COVID. Comparisons, when performed, were reported using χ² tests, independent samples median tests or t-tests according to the variable type and were accompanied by descriptive statistics.^11^,^13^ For the 10 most frequently reported symptoms, counts of how many times each pair of symptoms were reported by a participant were calculated to obtain symptom co-occurrence matrices for ME/CFS and long COVID. Hierarchical clustering analysis (HCA) was then performed using the Euclidean distance method to obtain distance matrices, with the complete agglomeration method used for clustering (default settings in the heatmap.2 function in R V.4.3.2).^14 15^ HCA was conducted using symptom co-occurrence matrices as input. Symptom co-occurrence matrices were constructed by calculating the frequency with which pairs of symptoms were reported together. Clustering was performed using the Euclidean distance metric to compute dissimilarities and the complete agglomeration method for grouping. Clusters were visualised using dendrograms to highlight relationships among symptoms. Graph theory was applied to provide a further comprehensive analysis of symptom interactions. For graph theory analysis, symptom co-occurrence matrices were converted into weighted, undirected graphs where nodes represented symptoms and edges indicated co-occurrences. The strength of each edge was determined by the frequency of co-occurrence. Metrics such as degree centrality (to identify the most connected symptoms), betweenness centrality (to determine symptoms acting as bridges) and clustering coefficients (to measure the tendency of symptoms to form clusters) were calculated using Python libraries. Network graphs were visualised to interpret the relationships between symptoms. The strength of each edge was determined by the frequency of co-occurrence, forming weighted networks for both ME/CFS and long COVID. Key graph metrics were calculated: degree centrality to identify the most connected symptoms, betweenness centrality to determine symptoms that act as bridges within the network and clustering coefficient to measure the tendency of symptoms to cluster together. Data analysis was completed using SPSS V.29.0 (SPSS, Chicago, Illinois, USA), Python V.3.12 with the PyCharm IDE 2024.4.1 (JetBrains) and R. Statistical significance was indicated at p<0.05.^16 17^

### Patient and public involvement

Patients and the public were actively involved in the development of this study. The survey was designed in consultation with individuals living with ME/CFS and long COVID, as well as with representatives from the ME Association, to ensure that the questions addressed the key concerns and experiences of those directly affected by these conditions. Feedback was gathered during the survey design phase through a pilot test, where individuals with ME/CFS trialled the survey and provided suggestions to improve its accessibility and relevance.

## Results

### Objective 1

#### Demographics

Participants: 10 458 people responded to the survey. 8804 confirmed that they or a close friend/family member had a diagnosis or symptoms of ME/CFS or long COVID. Of the participants with ME/CFS or long COVID, a subset (n=791) reported both conditions. These cases were analysed separately to explore potential differences in symptom clustering, healthcare experiences and self-management strategies. Of those linked to ME/CFS or long COVID, 7259 responded to gender and age questions. The demographics for age, gender and ethnicity are displayed in online supplemental tables 1,2 and 3 . 83.4% (6053) of these respondents were female, 14.9% (1077) were male and 1.8% (129) were non-binary or preferred to self-describe.

Diagnosis time for ME/CFS: the highest number of diagnoses (1664 individuals, 22.10%) occurred 1–2 years after noticing symptoms and informing a general practitioner (GP), with a number (1419, 18.84%) diagnosed within 7–12 months. 1240 individuals (16.47%) received their diagnosis within 3–6 months, while 975 (12.9) were diagnosed after more than 10 years. Additionally, 883 individuals (11.72%) were diagnosed within 3–4 years, with 461 (6.12%) and 388 (5.15%) individuals diagnosed within 5–6 years and less than 3 months, respectively. Fewer individuals were diagnosed within 7–8 years (249, 3.31) and 9–10 years (201, 2.67%), and 51 (0.68%) individuals chose not to disclose their diagnosis time (online supplemental table 4).

#### NHS experience after being diagnosed

NHS experience after being diagnosed with ME/CFS, 2157 respondents (33.76%) described their experience as very poor, poor and mixed experiences were also commonly reported, with 18.39% (1175) and 25.52% (1631) of respondents, respectively. Only 338 respondents reported having good experiences (5.29%), and even fewer rated their experience as excellent (89, 1.39%) or did not know (34, 0.53%).

**Figure 1:**
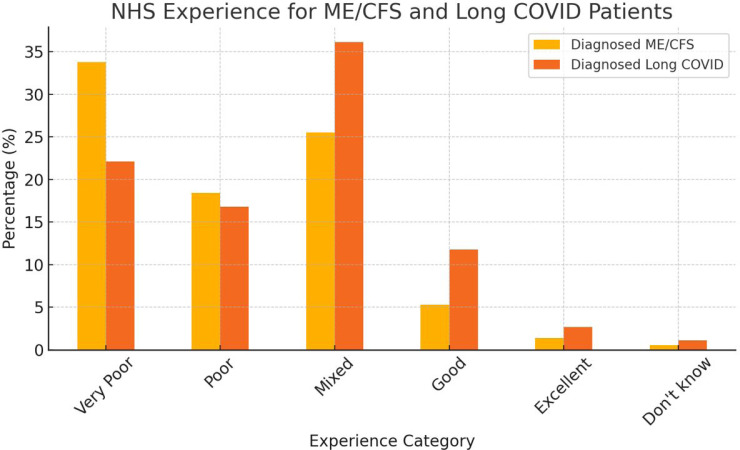
NHS experience and specialist visits. This figure presents responses regarding NHS experiences and access to specialist care for individuals with ME/CFS and long COVID. It categorises overall satisfaction with NHS services (very poor, poor, mixed, good, excellent) and illustrates the frequency of specialist visits, highlighting challenges in accessing specialised care. ME/CFS, myalgic encephalomyelitis/chronic fatigue syndrome; NHS, National Health Service.

Diagnosed long COVID: the survey on long COVID diagnosis revealed that a significant portion of respondents (258, 36.13%) had mixed feelings about their experience with the NHS. Furthermore, 22.13% (158) and 16.81% (120) of respondents reported very poor and poor experiences, respectively. On the positive side, 11.76% (84) of respondents reported good experiences, with 19 rating their experience as excellent (2.66%) or expressing uncertainty (8, 1.12%) (figure 1, online supplemental table 5).

#### Specialist visit

Many respondents (1255, 43.40%) had not seen a specialist in 5 years or longer. 16.32% (472) were last seen 3–4 years ago, 12.07% (349) were last seen 2–6 months ago, 11.10% (321) were last seen 1–2 years ago and 8.89% (27) were last seen in the last month. A very small percentage of respondents stated that they had not been seen (78, 2.70%) or did not know (13, 0.45%). Regarding the timing of their last specialist visit (Long Covid clinic), the majority of respondents (133, 37.46%) were last seen 2–6 months ago, while 23.66% (84) were last seen in the last month. Additionally, 16.90% (60) were last seen 1–2 years ago, and 12.96% (46) were last seen 7–11 months ago. A small fraction of respondents have not been seen (24, 6.76%) or expressed uncertainty about their last visit (3, 0.85%) (online supplemental table 6).

**Figure 2:**
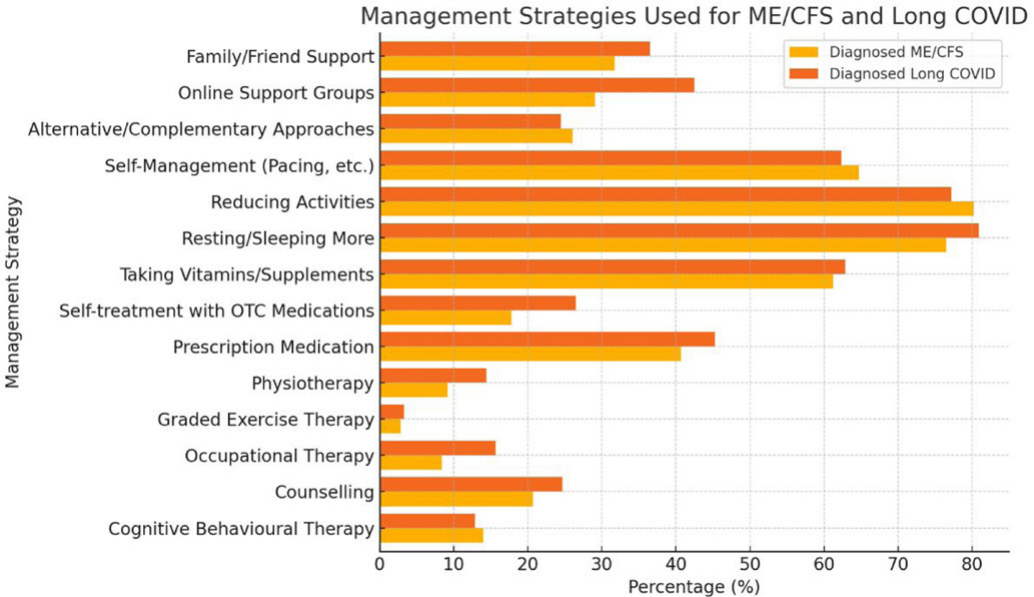
Support systems and management strategies. This figure summarises the various support systems accessed by individuals with ME/CFS and long COVID, including NHS services, online support groups and private healthcare providers. It also highlights the strategies used for symptom management, such as pacing, medication, self-management approaches and alternative therapies. ME/CFS, myalgic encephalomyelitis/chronic fatigue syndrome.

Management: people with ME and long COVID use similar strategies to manage their symptoms (figure 2). People with ME: the majority of diagnosed patients reported having to reorganise their life and take things a lot easier (4939, 80.8%), rest and sleep a lot more than before (4678, 76.5%) and reduce all activities (4908, 80.2%). A significant number of diagnosed patients also reported trying to balance activities with rest (4382, 71.6%), taking vitamins or supplements (3740, 61.2%) and using self-management approaches (3958, 64.7%). Among undiagnosed patients, the majority reported having to reorganise their life and take things a lot easier (223, 79.4%), rest and sleep a lot more than before (221, 78.6%) and reduce all activities (227, 80.8%). In people with long COVID, the majority of diagnosed patients also reported having to reorganise their lives and make things a lot easier (567, 83.3%), rest and sleep a lot more than before (550, 80.8%) and reduce all activities (528, 77.5%). A significant number of diagnosed patients also reported trying to balance activities with rest (465, 68.3%), taking vitamins or supplements (434, 63.7%) and using self-management approaches (433, 63.6%). Among undiagnosed patients, the majority reported having to reorganise their life and take things a lot easier (55, 71.4%), rest and sleep a lot more than before (63, 81.8%) and reduce all activities (57, 74.0%).

Management and support were reported for people with ME, with 1096 of diagnosed patients having received an assessment from an ME/CFS specialist service (20.2%) in the last 2 years and accessing information and support from a specialist (1092, 20.1%). However, most diagnosed patients said they had received no help from the NHS in the last 2 years (2864, 52.8%). Fewer patients received a diagnosis from a GP (754, 13.9%), an assessment from a consultant (477, 8.8%) or an assessment from a GP (495, 9.1%). However, in people with long COVID, most diagnosed patients received an assessment from a long COVID clinic (405, 62.6%) in the last 2 years. Many diagnosed patients also received a diagnosis from a GP (392, 60.6%) and information and support from a specialist (304, 47.0%). Fewer patients received an assessment from a GP (190, 29.4%), a consultant (153, 23.6%) or information and support from a GP (81, 12.5%) (online supplemental table 7).

**Figure 3:**
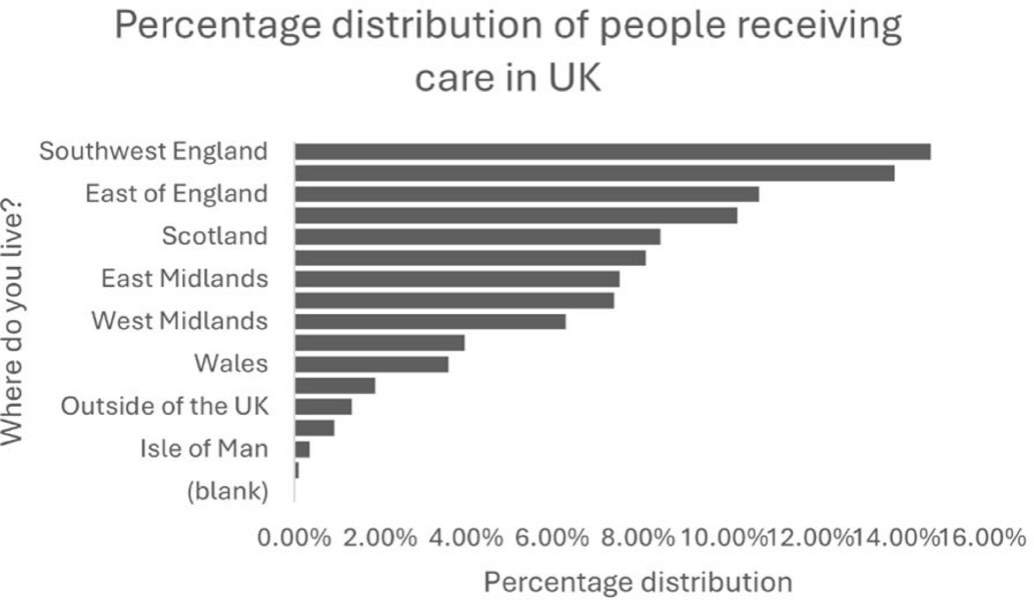
Geographical distribution of myalgic encephalomyelitis/chronic fatigue syndrome (ME/CFS) patients receiving care. This figure maps the regional distribution of individuals receiving care for ME/CFS across the UK, identifying variations in healthcare access and the geographic concentration of patients receiving specialist services.

People with ME: figure 3 shows that nearly half of the diagnosed patients (3026, 47.6%) were seen in a hospital-based ME/CFS specialist service. NHS GPs saw most diagnosed (4758, 74.9%) and undiagnosed (177, 63.0%) patients. The ME Association was a significant source of support for both diagnosed (3497, 55.0%) and undiagnosed (77, 27.4%) patients. Many patients also sought information online (3709, 58.4% diagnosed; 183, 65.1% undiagnosed).

For people with long COVID: most diagnosed patients (371, 52.3%) were seen in a hospital-based long COVID clinic. NHS GPs saw the highest number of both diagnosed (582, 82.1%) and undiagnosed (47, 61.0%) patients. A significant number of patients also sought information online (390, 55.0% diagnosed; 38, 49.4% undiagnosed) and from family and friends (260, 36.7% diagnosed; 27, 35.1% undiagnosed), figure 3.

Social care: the highest percentage of participants with ME and long COVID receiving care in this study were from South West England at 14.80% (1081). This is closely followed by South East England at 13.97% (1020), East of England at 10.80% (789) and Northwest England at 10.31% (753). Other regions such as Scotland, Yorkshire and the Humber, East Midlands, London and West Midlands have percentages ranging from 6.31% to 8.52%. The regions with the lowest percentage of people are the Isle of Man, at 0.34% (25), and the Channel Islands, at 0.10% (7), figure 3.

Impact on life: people with ME/CFS condition: among diagnosed patients, the majority (3709, 58.04%) reported a moderate impact on their lives, followed by a mild impact (1423, 22.27%) and a severe impact (1121, 17.54%). Among undiagnosed patients, the majority reported a moderate impact (159, 55.99%), followed by a mild impact (101, 35.56%) and a severe impact (22, 7.75%). Impact of long COVID: among diagnosed patients, the majority (404, 56.58%) reported a moderate impact on their lives, followed by a mild impact (193, 27.03%) and a severe impact (92, 12.89%). Among undiagnosed patients, the majority also reported a moderate impact (46, 59.74%), followed by a mild impact (22, 28.57%) and a severe impact (8, 10.39%).

### Objective 2

**Figure 4:**
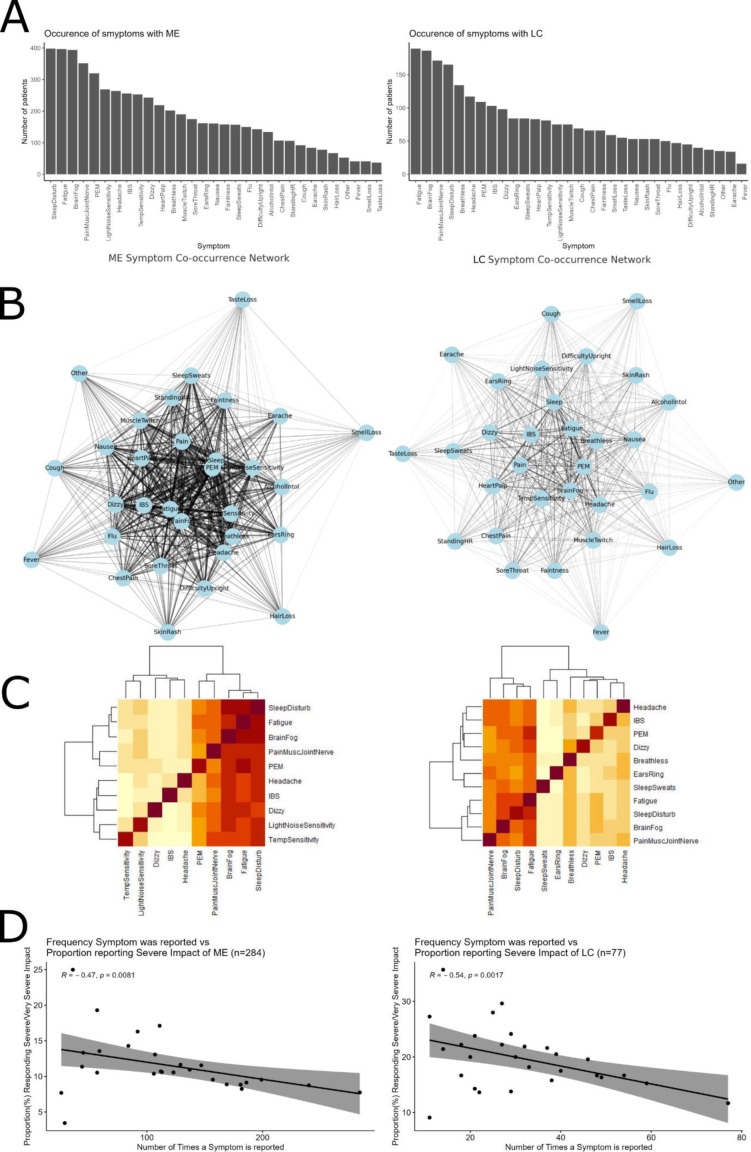
Symptom prevalence and co-occurrence patterns. This figure presents data on the occurrence and relationships of symptoms in myalgic encephalomyelitis (ME)/chronic fatigue syndrome and long COVID. (A) Most commonly reported symptoms for both conditions. (B) Graph theory visualisation of symptom co-occurrence networks, identifying relationships between frequently reported symptoms. (C) Heatmap of symptom co-occurrence, with darker shades indicating stronger associations between symptoms. (D) Correlation between symptom prevalence and reported severity of impact, illustrating how certain symptoms contribute to higher levels of disability. IBS, irritable-bowel-type symptoms; PEM, postexertional malaise.

Symptom prevalence with ME/CFS: a total of 450 participants within the subset of those diagnosed with ME/CFS responded with information about symptom presentation (Q5=diagnosis, Q8=symptom) (figure 4A). The 10 most commonly reported symptoms with ME/CFS (starting with the most commonly reported) were unrefreshing sleep or sleep disturbance (n=398, 88.22%), debilitating fatigue/exhaustion (n=397, 71.11%), brain fog or cognitive difficulties (n=394, 88.44%), pain in muscles, joints or nerves (n=352, 87.65%), PE M (n=320, 78.22%), increased sensitivity to light and noise (n=269, 58.67%), headache (n=264, 59.78%), irritable-bowel-type symptoms (IBS) (n=256, 54.00%), increased sensitivity to hot and cold temperatures (n=253, 56.22%) and dizziness (n=243, 56.89%).

Symptom prevalence with long COVID: there was a total of 267 participants within the subset of those diagnosed with long COVID that responded with information about symptom presentation (Q5=diagnosis, Q8=symptom) (figure 4A). The 11 (there were two in joint 10th position) most commonly reported symptoms with long COVID (starting with the most commonly reported) were debilitating fatigue/exhaustion (n=189, 42.00%), brain fog or cognitive difficulties (n=186, 41.33%), pain in muscles, joints or nerves (n=171, 38.00%), unrefreshing sleep or sleep disturbance (n=165, 36.66%), breathlessness (n=134, 29.78%), headache (n=117, 26.00%), PEM (n=109, 24.22%), IBS (n=103, 22.89%), dizziness (n=98, 21.78%), ringing in the ears (n=84, 18.67%) and excessive sweating when asleep (n=84, 18.67%).

Most reported symptoms with ME/CFS or long COVID taken together: of the 10 most prevalent symptoms for ME/CFS and long COVID, 8 were common to both: brain fog, dizziness, fatigue, headache, IBS, pain in muscles, joints or nerves, PEM and sleep disturbances. Light/noise sensitivity and temperature sensitivity were only within the 10 most common symptoms with ME/CFS, and breathlessness, ears ringing and sleep sweats were only within the 10 most common symptoms with long COVID.

Symptom co-occurrence network for individuals with ME (figure 4B): this network uses graph theory to visualise how symptoms reported by the survey respondents inter-relate, based on their co-occurrence: nodes represent different symptoms experienced by individuals. Edges indicate that these symptoms frequently co-occur. The thickness of an edge correlates with the frequency of co-occurrence; thicker edges mean that the symptoms appear together more often among respondents. The network layout is structured so that symptoms with more connections are generally positioned more centrally, suggesting they may be common or pivotal symptoms in the condition.

The network graph visually represents how different symptoms interconnect based on their co-occurrence in patient reports. Nodes in the network represent various symptoms, and edges between them indicate that these symptoms are frequently experienced together by individuals with ME. Thicker edges denote a higher frequency of co-occurrence, highlighting symptom pairs or clusters that are more commonly reported.

This network analysis shows that certain symptoms such as fatigue, pain and brain fog not only show strong connections to many other symptoms but also feature centrally within the network. This suggests that these symptoms are particularly prevalent and might be central to the experiences of many individuals with ME. Additionally, severity analysis indicates that these symptoms are often associated with higher impact severity scores, suggesting they significantly affect patients’ quality of life.

Symptom co-occurrence network for individuals with long COVID (figure 4B): a symptom co-occurrence network was constructed using graph theory to understand the relationships between different symptoms of long COVID and their co-occurrence patterns.

The graph was created by treating each symptom as a node. Edges between nodes were drawn if the corresponding symptoms co-occurred in at least one individual, and the weight of each edge was proportional to the number of individuals in which both symptoms appeared together. Visualising this network provided a graphical representation of how symptoms cluster together.

Symptoms such as fatigue, brain fog, and breathlessness tend to be linked with higher severity ratings. These symptoms, represented by nodes connected with thicker edges in the network, signify common co-occurrence and a stronger impact on the individuals’ health.

Associations of symptoms: for both ME/CFS and long COVID, we explored co-occurrence (how often a participant reported both of a pair of symptoms) within the 10 most frequently reported symptoms, then used HCA to identify symptom clusters.

ME/CFS symptom clusters: there were 448 participants with a diagnosis who responded to relevant questions (Q5=diagnosis, Q8=symptoms) and who reported at least one of the 10 most common symptoms. There were three clear clusters of co-occurrence for ME/CFS (figure 4C).

Cluster 1: debilitating fatigue/exhaustion, unrefreshing sleep or sleep disturbance, and ‘brain fog’ or cognitive difficulties.

Cluster 2: PEM, and pain in muscles, joints or nerves.

Cluster 3: headache, increased sensitivity to light and noise, dizziness, increased sensitivity to hot and cold temperatures, and IBS.

Long COVID symptom clusters: there were 261 participants with a diagnosis who responded to relevant questions (Q5=diagnosis, Q8=symptoms) and who reported at least one of the 10 most common symptoms. There were three clear co-occurrence clusters with long COVID (figure 4C).

Cluster 1: debilitating fatigue/exhaustion, ‘brain fog’ or cognitive difficulties, pain in muscles, joints or nerves, and unrefreshing sleep or sleep disturbance.

Cluster 2: ringing in the ears and excessive sweating when asleep.

Cluster 3: breathlessness, headache, PEM, IBS and dizziness.

Symptoms and impact of condition: we explored the relationship between the impact of the condition (ME/CFS or long COVID) and the prevalence of symptoms within the subset of participants with a diagnosis who responded to relevant questions (Q5=diagnosis, Q8=symptoms, Q15b=impact severity). With both ME/CFS and long COVID, the more prevalent a symptom was, the lower the proportion (%) of respondents with that symptom that reported ‘severe’ or ‘very severe impact’ of the condition. For example, rarer symptoms are associated with more severe impact on life (figure 4D).

### Objective 3

A total of 2892 cases of diagnosed ME/CFS were reported. The distribution of these cases over time is as follows: 8.89% of the cases were diagnosed in the last month. 12.07% were diagnosed 2–6 months ago. 5.08% were diagnosed 7–11 months ago. 11.10% were diagnosed 1–2 years ago. 16.32% were diagnosed 3–4 years ago. A significant proportion (43.40%) were diagnosed 5 years ago or longer. 2.70% of the respondents reported that they have not been seen. 0.45% of the respondents reported not knowing when they were diagnosed.

We found that the number of comorbidities was significantly related to time to diagnosis in people with ME/CFS, whereas this was not the case in people with long COVID. Other factors such as age, ethnicity and gender were not significantly related to time to diagnosis in participants with either ME/CFS or long COVID, R<0.1 and p>0.05.

### Objective 4

**Figure 5:**
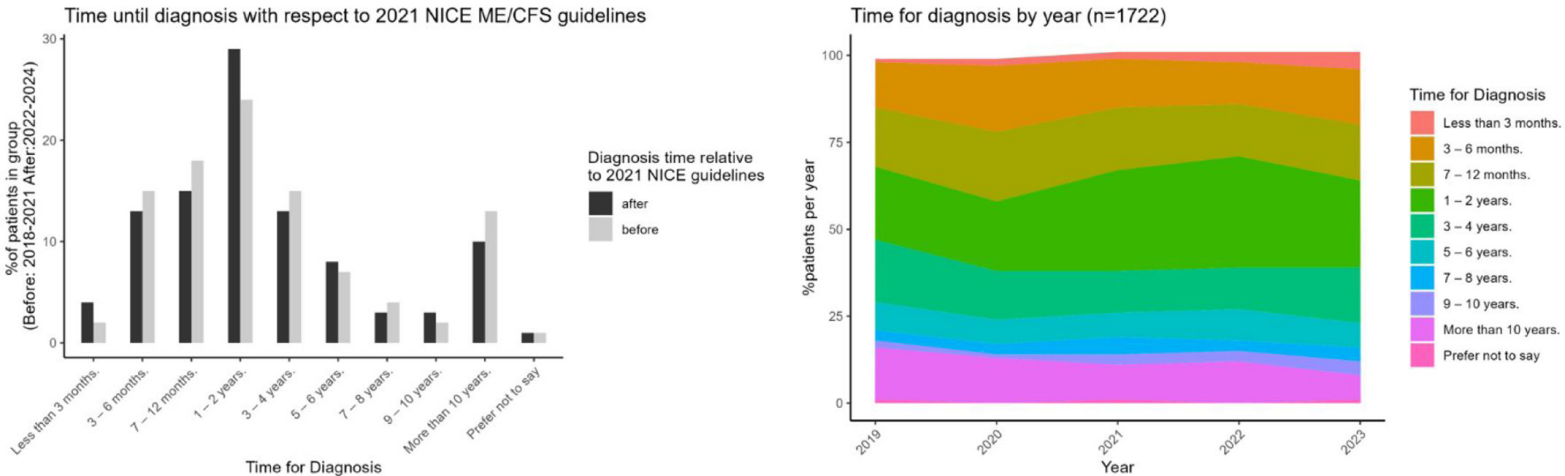
Time to diagnosis for ME/CFS. This figure explores the time taken to receive an ME/CFS diagnosis. Left: comparison of time to diagnosis before and after the 2021 NICE ME/CFS guidelines, assessing whether updated recommendations influenced diagnosis timelines. Right: historical trend analysis of ME/CFS diagnoses over the years, showing variations in diagnosis rates and potential improvements in early detection. ME/CFS, myalgic encephalomyelitis/chronic fatigue syndrome; NICE, National Institute for Health and Care Excellence.

We observed similar time to diagnosis in the first 12 months and in general for those diagnosed 3 years before (n=979) and after (n=743) the 2021 NICE guideline (figure 5, left).

Figure 5 (right and left) shows a potential trend to faster diagnosis in the 3-, 3 to 6- and 7 to 12-month periods after 2022, although trends were fluctuating over the reporting periods.

### Objective 5

To determine the impact of the NICE guideline on people with ME/CFS’s life and service experience since its publication in October 2021. According to this study result, 80 (10.1%) respondents from 4761 thought that the NICE guideline’s recommendations had made a positive difference to the healthcare they received, with 70 (1.5%) people noting a positive significant impact.

480 (10.1%) people from 4761 respondents thought the NICE guideline’s recommendations had made a positive difference to the healthcare they received, with 70 (1.5%) people noting a positive significant impact.

## Discussion

This study reports on the largest UK survey undertaken by the MEA, which has been reporting on symptoms, diagnosis and management for people with ME/CFS since the publication of the 2021 NICE guideline.^3^ Importantly, the survey reports on both ME/CFS and long COVID. Based on the findings of this study, only a small number of 6.9% of participants with ME/CFS and 14.42% with long COVID rated their experience with the NHS as good to excellent, with the remainder reporting poor, very poor or mixed experiences. Our findings suggest a need to consider how the NHS’s handling of long COVID and ME/CFS could be enhanced to support people with this condition better. The data highlights the severity of the impact of both long COVID and ME/CFS significantly impacting people’s lives, with the majority of patients experiencing a moderate to severe impact, requiring them to make substantial changes to their lifestyle and daily activities. It also highlights the importance of self-management approaches and strategies.^18^ Importantly, we find symptom clusters and co-occurrences of symptoms that underscore potential directions for further investigation that may help improve the time to diagnosis and provide key service requirements and associated team members for services for people with both ME/CFS and long covid. Additionally, the survey results indicate that individuals diagnosed with ME/CFS are less likely to have recent interactions with specialists compared with those diagnosed with long COVID. Our observations suggest there is a need for better access for both with possible potential for interaction of services, considering the similar symptoms, although the presentation of symptoms may differ.^19^ Our findings suggest that good evidence, key self-management information for lifestyle, activity, sleep and symptom management should be made available to benefit people with both conditions through both NHS services and online media.

According to this study, we noted for many a difficulty in establishing a diagnosis, with the time of diagnosis for ME/CFS varying widely. The results confirmed that many people with ME/CFS are experiencing a serious and potentially harmful delay in having their diagnosis confirmed. Many individuals are diagnosed within the first 2 years, but a considerable number are diagnosed after more than 10 years. A larger proportion of ME/CFS cases were diagnosed 5 years ago or longer, compared with long COVID cases, which are more recent. This is likely due to the recent onset of the COVID-19 pandemic. Of note, in ME/CFS, a high number of symptoms was associated with a longer time to diagnosis. This could indicate challenges in diagnosing this condition and is in line with previous observations.^20^ Considering that diagnosed individuals reported accessing more services, we propose a need for research to improve precise and timely diagnosis.^21^

This study demonstrated that a range of specialist services, such as the ME Association, private GPs and healthcare professionals (HCPs), online resources, and support groups, are also important in supporting patients.^22^ This observation is important considering the push towards online support and the use of evidence-based hybrid technologies.^23^ We further observed the need for involvement across the disciplines of HCPs at a time of UK shortages, as highlighted by the 2030 WHO rehabilitation strategy.^24^ The perceived lack of service provision suggests the need for innovation, as suggested by the WHO, for more use of technology and specialist training to deliver optimal care more widely.^17^ This, alongside the reported use of online services, suggests that evidenced, key self-management information for lifestyle, activity, sleep and symptom management should be made available through both NHS services and online media.

The complexity of diagnosis for people living with ME/CFS has previously been highlighted. Here, we confirm the difficulty with diagnosis and found no change in the reported time to diagnosis before and after the publication of the NICE guideline. A key finding is that the number of comorbidities that individuals presented with was a significant factor in how long participants were diagnosed. Our findings support that the complexity of presentation may be a key factor in the timely diagnosis of people with ME/CFS. This observation aligns with one in three adults now living with more than one condition, with needs not met by established services^25^ and the urgent need to find new approaches to diagnose and subsequently manage people living with more than one condition and complex health conditions.^10^

When considering managing complexity in health,^26^ we observed three distinct symptom clusters with similar co-occurrence frequency for both ME/CFS and long COVID. For ME/CFS, cluster 1 (Fatigue, SleepDisturb, BrainFog) included two of the four most reported symptoms for ME/CFS (range in number of reports for symptoms in cluster 1: 320–398). The symptoms in cluster 1 were most often reported alongside other symptoms in cluster 1, followed by cluster 2 and cluster 3. Cluster 2 (PEM, PainMuscJointNerve) included two of the four most commonly reported symptoms for ME/CFS (range: 352–394). Symptoms in cluster 2 were more often reported alongside symptoms in cluster 1 than with each other, which occurred at a similar frequency as with symptoms in cluster 3. Symptoms in cluster 3 (Headache, LightNoiseSensitivity, Dizzy, TempSensitivity, IBS) were all reported less frequently than those in cluster 1 or cluster 2 (range: 243–269). All symptoms in cluster 3 showed similar patterns of co-occurrence, most often reported alongside symptoms in cluster 1, followed by cluster 2, then cluster 3.

For long COVID, cluster 1 (Fatigue, Brain Fog, Pain, MuscJointNerve, Sleep Disturb) included the four most reported symptoms with long COVID (range: 165–189). The symptoms in cluster 1 were most often reported alongside other symptoms in cluster 1, followed by symptoms in cluster 3, and then symptoms in cluster 2. Cluster 2 (EarsRing, SleepSweats) included the two least commonly reported symptoms with long COVID (both reported 84 times). Symptoms in cluster 2 were reported at a similar low frequency with all other symptoms within the 10 most reported symptoms for long COVID. Cluster 3 (Breathless, Headache, PEM, IBS, Dizzy). All symptoms were reported at a moderate frequency out of the 10 most reported symptoms (range: 98–134). All symptoms in cluster 3 showed similar patterns of co-occurrence, most often reported alongside symptoms in cluster 1, followed by cluster 3, then cluster 2. Further observation through graph theory analysis shows co-occurrence in pairs of symptoms, with some symptoms less likely to occur and the most common symptoms more likely to occur with more severe disease impact.

The results presented above provide an overview of how often pairs of symptoms co-occur with each other and potential groupings that may inform future research into precision treatments.^27^ These results provide an interesting insight into how the most frequent complaints of patients relate to each other. However, it should be considered that this analysis cannot account for relationships outside of a pairwise relationship and, therefore, cannot be used to build profiles of mutually inclusive or exclusive symptom clusters, which should be a goal for future work. Indeed, the consideration alongside the coexistence of multiple conditions would be an important area for future work and highlights that management of ME/CFS may be better understood within this context.^28^ Finally, and significantly, we were surprised to find that a small but notable percentage of individuals who reported a diagnosis of ME/CFS did not experience PEM, which is a key diagnostic characteristic of the condition. This observation underscores the potential challenges in using terms such as PEM. It is possible that some individuals do experience PEM but may not fully comprehend the term, or it could be that some people are misdiagnosed with ME/CFS when they have an alternative explanation for their chronic fatigue. This could be due to some doctors not being fully informed about the current criteria requiring the presence of PEM for a diagnosis of ME/CFS, or it could be that some of the older diagnostic criteria for ME/CFS do not mandate the presence of PEM.

### Strengths and limitations

This study has several limitations. First, the use of a convenience sample relying on self-reported eligibility and data introduces potential biases, including selection and reporting biases. Our sample may overrepresent individuals who are more engaged with patient advocacy groups or have access to online platforms, potentially excluding those with limited internet access or differing healthcare experiences. Our sample comprised 6053 females, 1077 males, 129 non-binary individuals and 3155 participants who preferred not to disclose their gender, spanning all four nations of the UK. While this demographic distribution reflects the typical population engaged with ME/CFS and long COVID services, it may not represent the broader patient population, limiting our findings’ generalisability. Additionally, the reliance on self-reported diagnoses without verification through clinical records or standardised diagnostic tools may affect the reliability of the data, as self-reported symptoms can lead to both overestimation and underestimation of disease prevalence. However, the substantial sample size of 8804 participants enhances the study’s statistical power, providing a margin of error between 1% and 1.5% at a 99% CI. Including caregivers reporting on behalf of individuals with ME/CFS and long COVID may have introduced variability in responses; nonetheless, sensitivity analyses indicated no significant differences, supporting the inclusion of perspectives from those with severe disease, as recognised in the NICE guidelines. The study offers a comprehensive analysis of the survey findings by using all available data without excluding incomplete responses. However, this approach may introduce variability, as participants who did not complete all sections of the survey may systematically differ from those who did. The distinction between ME/CFS (ICD-10 G93.31) and long COVID (ICD-10 U09.9) is complex and may not always be clearly understood by patients or clinicians. This limitation is particularly relevant in cases of post-COVID-19 fatigue where diagnostic ambiguity may arise. While our survey allowed participants to select multiple diagnoses, self-reporting may not capture nuances in clinical criteria, and future studies could benefit from incorporating clinician-verified diagnoses or more specific diagnostic questions to address this overlap. Finally, data collection occurred in the UK during a period of notable dissatisfaction with NHS service provision, as highlighted in the 2023 British Social Attitudes survey, which may have influenced participants’ responses and should be considered when interpreting the conclusions.

## Conclusions

ME/CFS impacts thousands of people daily. Nearly 70% of sufferers have moderate or severe symptoms requiring them to make substantial changes to their lifestyle and daily activities. A key concern was the observed very poor times for referral for diagnosis, poor diagnosis times, poor referrals to specialist services and no significant improvements since the publication of the NICE guidelines. There is a significant complexity of co-occurring conditions, which correlate to a longer time needed to reach a diagnosis. The complexity of co-occurring conditions may extend the time for a diagnosis even further, possibly further impacted by the symptom complexity and variability. A potential target to substantially improve experiences is through the current NHS and social care services, with key areas suggested as more equitable access, better-coordinated services, comprehensive provision, and support to align with NICE guidelines across all four nations and regions. There is significant overlap in the experiences of people with long COVID and ME/CFS from symptoms to self-management, NHS care provision and access to social care. The high number of COVID-19 outbreaks and high numbers of people living with ME/CFS and long COVID may present a ticking time bomb for the NHS and social care services, and an urgent need for education of all health professionals and social care staff on the diagnosis, care and management of people with ME/CFS, especially those with severe ME/CFS.

We observed symptom cluster associations and co-occurrences that may inform future research into more targeted interventions. Finally, we propose a number of possible areas for further research, including the need to develop diagnostic measures, measures for monitoring systems and interventions for management. Areas that may be a focus for research could explore the specific issues faced by patients and potential solutions, particularly around diagnosis and precision management, including evidenced systems for monitoring systems and interventions for self-management of lifestyle, activity, sleep and symptom management. The range of symptom management and treatment strategies were mainly self-administered (such as changes to day-to-day life and pacing) and, while highlighted as helpful, did not involve access to a multidisciplinary team of healthcare practitioners. We propose a need to establish the UK must establish a network of specialist referral services that follow NICE guideline recommendations on both staffing and the services they provide and access to social care throughout the UK.

## supplementary material

10.1136/bmjopen-2024-094658 online supplemental file 1

10.1136/bmjopen-2024-094658 online supplemental file 2

## Data Availability

Data are available upon reasonable request.
