# Supplementary material for: Understanding symptom clusters, diagnosis and healthcare experiences in myalgic encephalomyelitis/chronic fatigue syndrome and long COVID: a cross-sectional survey in the UK
Source: BMJ Open. 2025 Apr 2;15(4):e094658. doi: 10.1136/bmjopen-2024-094658 (PMC11966950; doi:10.1136/bmjopen-2024-094658)
Supplement: online supplemental file 1 [file bmjopen-15-4-s001.pdf]

The ME Association
Count ME In Survey
18 May 2023
Final: Version 9

<u>Introduction on first page of survey (longer intro on MEA website launch page):</u>

Thank you for taking the time to complete the survey today for the ME Association. It will take around 10 minutes, and if you aren't able to complete it all in one go, your progress will automatically save and you can return later for up to 3 days.

This survey is run by One Minute to Midnight, a market research agency, on behalf of the ME Association. All your answers will be treated in complete confidence in accordance with the Market Research Society Code of Conduct and GDPR. We will not be asking you for any personally identifiable information.

Completing this survey will enable the ME Association to:

1. Inform more people about ME/CFS and Long Covid, help them obtain an accurate diagnosis if needed, and find the right support to improve their life quality.
2. Produce an independent report that will raise awareness and help us improve the standard of healthcare by working with the NHS and social care services.

ME = Myalgic Encephalopathy or Encephalomyelitis.
CFS = Chronic Fatigue Syndrome
Long Covid = Post-Covid Syndrome

CLICK HERE TO START THE SURVEY

SECTION A: SCREENING

Q1. Do you, or any of your close friends or family currently have a diagnosis of any of the following? Or do you think you or a close friend or family member might have symptoms of any of the following?
Please tick all that apply

1. ME / CFS or Long Covid
2. Diabetes
3. Alzheimer's
4. Rheumatoid Arthritis
5. Multiple Sclerosis
6. Cancer
7. None of the above

Q2. Which one of these most applies to you?

1. I have symptoms and a diagnosis of ME/CFS
2. I have symptoms and a diagnosis of Long Covid
3. I think I have ME/CFS symptoms but have not been diagnosed
4. I think I have Long Covid symptoms but have not been diagnosed
5. I had symptoms or a diagnosis of ME/CFS but have now recovered
6. I had symptoms or a diagnosis of Long Covid but have now recovered
7. I have a close family member or friend that has been diagnosed with ME/CFS
8. I have a close family member or friend that has been diagnosed with Long Covid
9. I have a close family member or friend with symptoms of ME/CFS but no diagnosis
10. I have a close family member or friend with symptoms of Long Covid but no diagnosis
11. None of these

ASK IF FRIEND/FAMILY HAS ME/CFS/LC OR SYMPTOMS AND NOT THEMSELVES (Q2 = CODE 7, 8, 9 or 10)
Q3. This survey will ask about the experiences of people with (or who might have) ME/CFS or Long Covid. Do you have the permission of the friend or family member to complete this survey on their behalf? If not, please send them the survey or come back and complete the survey with them.

1. Yes
2. No

SHOW IF YES AT Q3:
Please answer the rest of the survey from the perspective of your friend or family member.

RESPONDENT TYPE:

- ME / CFS: Q2 = 1 or 7
- Long Covid: Q2 = 2 or 8
- Undiagnosed: Q2 = 3, 4, 9 or 10

UNDIAGNOSED ROUTE:

UNDIAGNOSED ONLY
Q4. Have you consistently experienced any of these symptoms for at least 3 months?
Please tick all that apply

1. Debilitating Fatigue / Exhaustion: Symptoms are worsened by activity and do not get better with rest.
2. Post-Exertional Malaise: Symptoms are made worse after activity, the impact is often delayed, is disproportionate to the activity and has a long recovery time.
3. Unrefreshing sleep or sleep disturbance: This may include feeling exhausted, flu-like, and stiff on waking, broken or shallow sleep, altered sleep patterns, or needing to sleep too much.
4. 'Brain Fog' or cognitive difficulties: This may include problems finding words or numbers, difficulty speaking, slowed responses, short-term memory problems, and difficulty concentrating.

[ME/CFS & LC Additional Symptoms]

5. Alcohol intolerance.

6. Difficulty maintaining an upright position.
7. Dizziness.
8. Excessive sweating when asleep.
9. Faintness.
10. Headache.
11. Increased sensitivity to hot and cold temperatures.
12. Increased sensitivity to light and noise.
13. Irritable-bowel-type symptoms.
14. Muscle twitches or jerks.
15. Nausea.
16. Pain in muscles, joints, or nerves.
17. Heart palpitations.
18. Flu-like symptoms (persistent).
19. Rapid rise in heart rate on standing up.

[LC Additional Symptoms]

20. Breathlessness.
21. Chest pains.
22. Cough.
23. Earache.
24. Fever.
25. Loss of taste.
26. Loss of smell.
27. Hair loss.
28. Ringing in the ears.
29. Skin rashes.
30. Sore throat.
31. Other (please specify): [ANCHOR] [OPEN END]
32. None of the above: [ANCHOR] [EXCLUSIVE] [**CLOSE**]
33. Prefer not to say: [ANCHOR] [EXCLUSIVE] [**CLOSE**]

ASK IF UNDIAGNOSED

Q5. You said you experience:

- Debilitating Fatigue / Exhaustion
- Post-Exertional Malaise
- Unrefreshing Sleep or Sleep Disturbance
- Brain Fog or cognitive difficulties

For how long have you experienced all of these symptoms?

1. Less than 6 months
2. 6-11 months
3. 1 – 3 years
4. 4 – 5 years
5. 6 – 7 years
6. 8 – 9 years
7. 10 years +
8. Don't know

ASK IF UNDIAGNOSED

Q6. What has prevented you from getting a diagnosis?
Please tick all that apply

1. I wasn't sure if my symptoms related to anything specific
2. I wasn't sure if my symptoms were related to ME/CFS or Long Covid
3. I wasn't able to get an appointment with a GP
4. I've been too unwell and can't get a GP to visit me at home
5. I am waiting for an appointment with a GP
6. I am waiting for a referral to a consultant
7. I am waiting for a referral to an ME/CFS specialist service
8. I am waiting for a referral to a Long Covid clinic
9. The GP didn't think my symptoms were related to ME/CFS or Long Covid
10. The GP didn't mention ME/CFS or Long Covid
11. The GP didn't believe ME/CFS or Long Covid were 'real'
12. The GP did not want to make a referral to a specialist
13. There are no ME/CFS specialist services in my area
14. There are no Long Covid clinics in my area
15. I don't think my symptoms are severe enough

16. I don't think a GP or consultant can help
17. I don't think an ME/CFS specialist service or Long Covid clinic can help
18. I have delayed making an appointment because I don't want to cause more work for the NHS
19. I will be making a GP appointment and seeking a diagnosis once I complete this survey
20. Other (please specify): [ANCHOR] [OPEN END]
21. None of the above: [ANCHOR] [EXCLUSIVE]
22. Prefer not to say: [ANCHOR] [EXCLUSIVE]

ASK IF WAITING FOR APPOINTMENT (Q10 = 5, 6, 7 OR 8)
Q7. For how long have you been waiting for an appointment or referral?

1. Less than a month
2. 1 – 3 months
3. 4 – 6 months
4. 7 – 12 months
5. 1-2 years
6. More than 2 years
7. Don't know

ASK ALL
Q8. SHOW TO ME/CFS: To what extent does ME / CFS impact your life today?
SHOW TO LC: To what extent does Long Covid impact your life today?
Please choose the one option that applies to you the most

1. **No Impact**
   You experience no problems with personal care or daily living. Your physical and cognitive abilities have been restored either completely or to a significant extent. You experience no symptoms at rest or after exertion and have not done so for 3 months or more. You feel capable of returning to full-time work or education and to leisure and social activities.
2. **Mild Impact**
   You are able to care for yourself and do some light domestic tasks (sometimes needing support), but you experience difficulties with mobility. You might still be able to work or go to school, college or university, but you have probably stopped all leisure and social activities. You often need reduced hours, take days off and use the weekend to cope with the rest of the week.
3. **Moderate Impact**
   You have reduced mobility and are restricted in all activities of daily living. You might have peaks and troughs in the range and severity of symptoms and your ability to initiate and complete activities. You have probably had to stop going work or school, college or university. You are likely to need rest periods, often in the afternoon. Your sleep at night is generally poor quality and disturbed [LC: and you may have difficulty breathing properly].
4. **Severe Impact**
   You are unable to do any activity for yourself or can carry out only minimal daily tasks (such as face washing or cleaning teeth). You have severe cognitive difficulties and may depend on a wheelchair for mobility. You are often unable to leave the house or have a severe and prolonged after-effect if you do. You may spend most of your time in bed [ME/CFS: and are often extremely sensitive to light and sound. / LC: and are likely to experience breathing problems and with heart function.]
5. **Very severe Impact**
   You are unable to leave your bed and are wholly dependent on the care and support of others. Physical or cognitive activities will be extremely limited. You need help with personal hygiene and eating [ME/CFS: and are very sensitive to light, noise, and touch / LC: You will experience severe difficulty breathing and with heart function]. You might not be able to swallow, and you may need to be tube-fed. You will be very weak and might be unable to communicate verbally.

Q9. Which of the following have you used to find help and support?
Please tick all that apply

1. An NHS counsellor or psychologist
2. An NHS GP
3. An NHS consultant
4. A pharmacist
5. Social care services
6. A private GP or consultant
7. A private counsellor or psychologist
8. The ME Association
9. Another UK charity
10. A non-UK charity

11. I searched online to get information
12. Family and friends
13. Other people with similar symptoms
14. I joined a local support group
15. I joined an online support group
16. Other (please specify): [ANCHOR] [OPEN END]
17. None of the above: [ANCHOR] [EXCLUSIVE]
18. Prefer not to say: [ANCHOR] [EXCLUSIVE]

ASK ALL

Q10. How do you manage your symptoms?
Please tick all that apply

1. I've had to reorganise my life and take things a lot easier. PIPE
2. I've had to reduce all activities. PIPE
3. I rest and sleep a lot more than before. PIPE
4. I try to balance activities with rest. PIPE
5. I've been too sick to be able to try anything.
6. I cope as best I can on my own.
7. I've been self-treating with over-the-counter medications (from a pharmacy). PIPE
8. I received medication from a GP (prescription). PIPE
9. I self-manage without the use of medications. PIPE
10. Physiotherapy PIPE
11. Occupational therapy PIPE
12. Graded Exercise Therapy PIPE
13. Counselling (to help me cope). PIPE
14. Cognitive behavioural therapy (to help me cope). PIPE
15. I've been using alternative or complementary approaches (massage, reiki, homeopathy, etc.). PIPE
16. I've been using self-management approaches (energy management, pacing, etc.). PIPE
17. I've been taking vitamins or supplements. PIPE
18. I've made changes to my diet. PIPE
19. Pulmonary rehabilitation PIPE
20. Other (please specify): [ANCHOR] [OPEN END] PIPE
21. None of the above: [ANCHOR] [EXCLUSIVE]
22. Prefer not to say: [ANCHOR] [EXCLUSIVE]

Q11. How helpful have each of these approaches been at helping you find relief from your symptoms?

1. Very Helpful.
2. Helpful.
3. Hard to tell.
4. Unhelpful.
5. Very unhelpful.
6. Prefer not to say: [ANCHOR] [EXCLUSIVE]

ME/CFS ROUTES: (Text substitutions used where appropriate)

ASK IF ME/CFS OR LONG COVID

Q4. How long did it take to receive a diagnosis of [ME/CFS / Long Covid] after you first noticed symptoms and informed a GP?

SINGLE CODE

1. Less than 3 months.
2. 3 – 6 months.
3. 7 – 12 months.
4. 1 – 2 years.
5. 3 – 4 years.
6. 5 – 6 years.
7. 7 – 8 years.
8. 9 – 10 years.
9. More than 10 years.
10. Prefer not to say

ASK IF ME/CFS OR LONG COVID

Q5. When were you diagnosed with [ME/CFS / Long Covid]?

Year:

ASK ME/CFS

Q6. Have you been diagnosed with any of the following conditions in addition to ME / CFS?

Please tick all that apply

1. Allergies.
2. Anxiety.
3. Chemical sensitivities.
4. Depression.
5. Diabetes (type 1).
6. Diabetes (type 2).
7. Endometriosis.
8. Fibromyalgia.
9. Hypermobility syndromes.
10. Incontinence.
11. Insomnia.
12. Interstitial cystitis.
13. Irritable bowel syndrome (IBS).
14. Mast Cell Activation Syndrome (MCAS).
15. Migraine.
16. Orthostatic Intolerance.
17. Postural Orthostatic Tachycardia Syndrome (PoTS).
18. Other (please specify):
19. None of the above:
20. Prefer not to say

ASK LONG COVID

Q7. Have you been diagnosed with any of the following conditions in addition to Long Covid?

Please tick all that apply

MULTICODE

1. Allergies.
2. Anxiety.
3. Arrhythmia.
4. Asthma.
5. Depression.
6. Diabetes (type 1).
7. Diabetes (type 2).
8. Eczema.
9. Endometriosis.
10. Heart failure.
11. Hypertension.
12. Inflammatory bowel disease (IBD).
13. Insomnia.
14. Incontinence.
15. Irritable bowel syndrome (IBS).
16. Migraine.
17. Myocardial Infarction.
18. Myositis.
19. Orthostatic Intolerance.
20. Postural Orthostatic Tachycardia Syndrome (PoTS).
21. Pulmonary Fibrosis.
22. Renal damage.
23. Sexual dysfunction.
24. Other (please specify):
25. None of the above:
26. Prefer not to say:

ASK ALL

Q8. SHOW TO ME/CFS: To what extent does ME / CFS impact your life today?
SHOW TO LC: To what extent does Long Covid impact your life today?
Please choose the one option that applies to you the most

1. **No Impact**
You experience no problems with personal care or daily living. Your physical and cognitive abilities have been restored either completely or to a significant extent. You experience no symptoms at rest or after exertion and have not done so for 3 months or more. You feel capable of returning to full-time work or education and to leisure and social activities.
2. **Mild Impact**
You are able to care for yourself and do some light domestic tasks (sometimes needing support), but you experience difficulties with mobility. You might still be able to work or go to school, college or university, but you have probably stopped all leisure and social activities. You often need reduced hours, take days off and use the weekend to cope with the rest of the week.
3. **Moderate Impact**
You have reduced mobility and are restricted in all activities of daily living. You might have peaks and troughs in the range and severity of symptoms and your ability to initiate and complete activities. You have probably had to stop going work or school, college or university. You are likely to need rest periods, often in the afternoon. Your sleep at night is generally poor quality and disturbed [LC: and you may have difficulty breathing properly].
4. **Severe Impact**
You are unable to do any activity for yourself or can carry out only minimal daily tasks (such as face washing or cleaning teeth). You have severe cognitive difficulties and may depend on a wheelchair for mobility. You are often unable to leave the house or have a severe and prolonged after-effect if you do. You may spend most of your time in bed [ME/CFS: and are often extremely sensitive to light and sound. / LC: and are likely to experience breathing problems and with heart function.]
5. **Very severe Impact**
You are unable to leave your bed and are wholly dependent on the care and support of others. Physical or cognitive activities will be extremely limited. You need help with personal hygiene and eating [ME/CFS: and are very sensitive to light, noise, and touch / LC: You will experience severe difficulty breathing and with heart function]. You might not be able to swallow, and you may need to be tube-fed. You will be very weak and might be unable to communicate verbally.

Q9. Which of the following have you used to find help and support for your symptoms or diagnosis?
Please tick all that apply

1. A NHS counsellor or psychologist
2. An NHS GP
3. An NHS consultant
4. A hospital-based ME/CFS specialist service – DIAGNOSED ME/CFS ONLY
5. A hospital based Long Covid clinic – DIAGNOSED LC ONLY
6. A pharmacist
7. Social care services
8. Private GP or consultant
9. A private counsellor or psychologist
10. The ME Association
11. Another UK charity
12. A non-UK charity
13. I searched online to get information
14. Family and friends
15. Other people with similar symptoms
16. I joined a local support group
17. I joined an online support group
18. Other (please specify): [ANCHOR] [OPEN END]
19. None of the above: [ANCHOR] [EXCLUSIVE]
20. Prefer not to say: [ANCHOR] [EXCLUSIVE]

ASK IF HAS USED NHS HELP
Q10a. What help have you received from the NHS in regard to ME/CFS or Long Covid in the last <u>2 years</u>?
Please tick all that apply

MULTICODE

1. An assessment from a GP.
2. An assessment from a consultant.
3. An assessment from an [ME/CFS specialist service / Long Covid clinic].
4. Information and support from a GP about learning how to live with [ME/CFS / Long Covid].

5. Information and support from a consultant about learning how to live with [ME/CFS / Long Covid].
6. Information and support from an [ME/CFS specialist service / Long Covid clinic] about learning how to live with the condition.
7. A diagnosis from a GP
8. None of the above:
9. Prefer not to say:

ASK IF HAS NOT USED NHS HELP (Q13 = NOT CODE ANY OF 1-5)
Q10b. Why have you not received help from the NHS in regard to ME/CFS or Long Covid in the last 2 years?

MULTICODE

1. I didn't know ME/CFS specialist services could help but will ask a GP after completing this survey.
2. I have not needed to use the NHS
3. I have not used an [ME/CFS specialist service / Long Covid clinic] because I didn't think they could help me.
4. I have not used an [ME/CFS specialist service / Long Covid clic] because there isn't one in my area.
5. I have not used the NHS. I paid for private healthcare.
6. I have not used the NHS. I don't believe they can help me.
7. None of the above: [ANCHOR][EXCLUSIVE]
8. Prefer not to say: [ANCHOR][EXCLUSIVE]

ASK IF HAS USED NHS HELP (Q13 = CODE ANY OF 1-5)
Q11. What is your overall experience of the NHS in regard to having ME/CFS or Long Covid?

1. Very poor
2. Poor
3. Mixed
4. Good
5. Excellent
6. Don't know

ASK IF CODE 1-5 AT Q15
Q12. Why do you say your experience of the NHS is [Q15 ANSWER]?
NON-MANDATORY OPEN END

ASK IF ME/CFS OR LONG COVID AND HAS USED SOCIAL CARE SERVICES (Q13 = 6)
Q13. What help have you received from social care services in regard to ME/CFS?
Please tick all that apply

1. I've had a care needs assessment that was successful.
2. I've had a care needs assessment that was unsuccessful.
3. I've not applied for an assessment but need social care support.
4. I am waiting to receive a care needs assessment.
5. I am waiting on the outcome of a care needs assessment.
6. I receive a personal budget and employ a carer.
7. I receive a personal budget and my local authority provide a carer.
8. I've had changes made to my home (e.g., grab rails, walk-in shower, etc.).
9. I need changes made to my home but haven't been offered any.
10. I've not needed changes made to my home.
11. I've been provided with equipment (e.g., wheelchair, personal alarm, etc.).
12. I need equipment but haven't been offered any.
13. I've not needed any equipment.
14. I've been moved to sheltered accommodation.
15. I've not been moved to sheltered accommodation, but I need to be.
16. I haven't needed support from social care services.
17. I didn't think I was eligible for social care but will contact my local authority after completing this survey.
18. None of the above:
19. Prefer not to say:

ASK IF ME/CFS OR LONG COVID AND HAS USED SOCIAL CARE SERVICES (Q13 = 6)

Q14. What is your overall experience of social care services in regard to having [ME/CFS / Long Covid]?

1. Very poor
2. Poor
3. Mixed
4. Good
5. Excellent
6. Don't know

ASK IF CODE 1-5 AT Q18

Q15. Why do you say your experience of social care services is..

NON-MANDATORY OPEN END

ASK IF ME/CFS OR LONG COVID AND HAS USED A SPECIALIST SERVICE / CLINIC (Q13 = 4)

Q16. You said you've used an [ME/CFS specialist service / Long Covid clinic]. Which of these apply to you in terms of the effect the service had on your health and wellbeing?
Tick all that apply

1. I received a medical assessment.
2. I received a diagnosis.
3. I felt my experiences were validated and I was made comfortable
4. I benefited from the information, support and management recommendations.
5. The healthcare professionals at the service were knowledgeable and understanding.
6. I felt the specialist service met my expectations.
7. I felt the service was able to tailor recommendations to meet my needs.
8. I felt the number of appointments with the service were sufficient
9. I was able to return to the service when needed e.g., when I relapsed.
10. I was able to access the service remotely when needed.
11. I benefited from the support when severely or very severely affected.
12. I helped to create a care and support plan which was shared with my GP.
13. None of these
14. Other. Please specify [ ]

ASK IF ME/CFS OR LONG COVID AND HAS USED A SPECIALIST SERVICE / CLINIC (Q13 = 4)

Q17. When were you last seen by [an ME/CFS specialist service / a Long Covid clinic]?

1. In the last month
2. 2 – 6 months ago
3. 7 – 11 months ago
4. 1 – 2 years ago
5. 3 – 4 years ago
6. 5 years ago or longer
7. I haven't been seen
8. Don't know

ASK IF ME/CFS OR LONG COVID AND HAS USED A SPECIALIST SERVICE / CLINIC (Q13 = 4)

Q18. What is the name [ME/CFS specialist service / Long Covid clinic] you have used?

NON-MANDATORY OPEN END

ASK IF ME/CFS OR LONG COVID AND HAS USED A SPECIALIST SERVICE / CLINIC (Q13 = 4)

Q19. What was your overall experience of this specialist service / Long Covid clinic?

1. Very poor
2. Poor
3. Mixed

4. Good
5. Excellent
6. Don't know

ASK IF CODE 1-5 AT Q23
Q20. Why do you say your experience of the specialist service is [Q23 ANSWER]?
NON-MANDATORY OPEN END

ASK ALL
Q21. How do you manage your symptoms?
Please tick all that apply

1. I've had to reorganise my life and take things a lot easier. PIPE
2. I've had to reduce all activities. PIPE
3. I rest and sleep a lot more than before. PIPE
4. I try to balance activities with rest. PIPE
5. I've been too sick to be able to try anything.
6. I cope as best I can on my own.
7. I've been self-treating with over-the-counter medications (from a pharmacy). PIPE
8. I received medication from a GP (prescription). PIPE
9. I self-manage without the use of medications. PIPE
10. Physiotherapy PIPE
11. Occupational therapy PIPE
12. Graded Exercise Therapy PIPE
13. Counselling (to help me cope). PIPE
14. Cognitive behavioural therapy (to help me cope). PIPE
15. I've been using alternative or complementary approaches (massage, reiki, homeopathy, etc.). PIPE
16. I've been using self-management approaches (energy management, pacing, etc.). PIPE
17. I've been taking vitamins or supplements. PIPE
18. I've made changes to my diet. PIPE
19. Pulmonary rehabilitation PIPE
20. Other (please specify):
21. None of the above:
22. Prefer not to say:

Q22. How helpful have each of these approaches been at helping you find relief from your symptoms?

7. Very Helpful.
8. Helpful.
9. Hard to tell.
10. Unhelpful.
11. Very unhelpful.
12. Prefer not to say: [ANCHOR] [EXCLUSIVE]

ASK ME/CFS OR LONG COVID
Q23. What improvements would you most like to see to the NHS that could improve your quality of life with regard to [ME/CFS / Long Covid]?

NON-MANDATORY OPEN END

ASK IF ME/CFS OR LONG COVID
Q24. **ME CFS:** A new NICE Guideline on ME/CFS was published in October 2021. This tells doctors how to treat / manage ME / CFS. Are you aware of this guideline?
Long Covid: The NICE Rapid Guideline on Long Covid was last updated in November 2021. This tells doctors how to treat / manage Long Covid. Are you aware of this guideline?

SINGLE CODE

1. Yes
2. No
3. Don't know

ASK IF AWARE OF GUIDELINE (Q27 = 1)

Q25. Do you think the NICE guideline's recommendations have had a positive effect on the healthcare that you have received?

1. Yes. It's made a big difference.
2. Yes. It's made some difference.
3. I don't know. I haven't used the NHS since it was published.
4. I don't know. I haven't read the Guideline.
5. No. I don't think the NHS is aware of the Guideline.
6. No. I haven't noticed a difference.
7. Don't know

ASK IF AWARE OF GUIDELINE (Q27 = 1)

Q26. Is there anything you'd like to tell us about your opinion of the NICE guideline?

NON-MANDATORY OPEN END

SECTION E: DEMOGRAPHICS (ASK ALL):

SHOW TO ALL: Thank you, for all your answers so far. The last few questions are just to find out a bit more about who has completed the survey.

ASK ALL

Q27. How old are you?

1. Under 18
2. 18 - 29
3. 30 – 39
4. 40 – 49
5. 50 – 59
6. 60 – 69
7. 70 – 79
8. 80 or older
9. Would rather not say

Q28. Which of the following best describes your gender?

1. Female
2. Male
3. Non-binary or prefer to self-describe
4. Would rather not say

Q29. Where do you live?

1. North East England
2. North West England
3. Yorkshire and the Humber
4. East Midlands
5. West Midlands
6. East of England
7. London
8. South East England
9. Southwest England
10. Wales
11. Northern Ireland
12. Scotland
13. Channel Islands

14. Isle of Man
15. Outside of the UK
16. Prefer not to say

Q30. In which county do you live?

[IF NORTH EAST ENGLAND]
1. Northumberland
2. Tyne and Wear
3. County Durham
4. Teesside

[IF NORTH WEST ENGLAND]
1. Cumbria
2. Lancashire
3. Greater Manchester
4. Merseyside
5. Cheshire

[IF YORKSHIRE AND THE HUMBER]
1. North Yorkshire
2. South Yorkshire
3. West Yorkshire
4. East Riding of Yorkshire
5. North Lincolnshire
6. North East Lincolnshire

[IF EAST MIDLANDS]
1. Derbyshire
2. Leicestershire
3. Lincolnshire
4. Northamptonshire
5. Nottinghamshire
6. Rutland

[IF WEST MIDLANDS]
1. Herefordshire
2. Shropshire
3. Staffordshire
4. Warwickshire
5. West Midlands
6. Worcestershire

[IF EAST OF ENGLAND]
1. Bedfordshire
2. Cambridgeshire
3. Essex
4. Hertfordshire
5. Norfolk
6. Suffolk

[IF SOUTH EAST ENGLAND]
1. Berkshire
2. Buckinghamshire
3. East Sussex
4. Hampshire
5. Isle of Wight
6. Kent
7. Oxfordshire
8. Surrey
9. West Sussex

[IF SOUTH WEST ENGLAND]
1. Bristol
2. Cornwall
3. Devon
4. Dorset
5. Gloucestershire
6. Somerset

7. Wiltshire

[IF WALES]
1. Blaenau Gwent
2. Bridgend
3. Caerphilly
4. Cardiff
5. Carmarthenshire
6. Ceredigion
7. Conwy
8. Denbighshire
9. Flintshire
10. Gwynedd
11. Isle of Anglesey
12. Merthyr Tydfil
13. Monmouthshire
14. Neath Port Talbot
15. Newport
16. Pembrokeshire
17. Powys
18. Rhondda Cynon Taf
19. Swansea
20. Torfaen
21. Vale of Glamorgan
22. Wrexham

[IF NORTHERN IRELAND]
1. Antrim
2. Armagh
3. Down
4. Fermanagh
5. Londonderry
6. Tyrone

[IF SCOTLAND]
1. Aberdeenshire
2. Angus
3. Argyll and Bute
4. Clackmannanshire
5. Dumfries and Galloway
6. Dundee
7. East Ayrshire
8. East Dunbartonshire
9. East Lothian
10. East Renfrewshire
11. Edinburgh
12. Falkirk
13. Fife
14. Glasgow
15. Highland
16. Inverclyde
17. Midlothian
18. Moray
19. Na h-Eileanan Siar (Western Isles)
20. North Ayrshire
21. North Lanarkshire
22. Orkney Islands
23. Perth and Kinross
24. Renfrewshire
25. Scottish Borders
26. Shetland Islands
27. South Ayrshire
28. South Lanarkshire
29. Stirling
30. West Dunbartonshire
31. West Lothian

[IF CHANNEL ISLANDS]
1. Jersey
2. Guernsey

3. Alderney
4. Sark

Q31. How would you describe your ethnicity?

White
1. English/Welsh/Scottish/Northern Irish/British
2. Irish
3. Gypsy or Irish Traveller
4. Any other White background

Mixed/Multiple ethnic groups
5. White and Black Caribbean
6. White and Black African
7. White and Asian
8. Any other Mixed/Multiple ethnic background

Asian/Asian British
9. Indian
10. Pakistani
11. Bangladeshi
12. Chinese
13. Any other Asian background

Black/ African/Caribbean/Black British
14. African
15. Caribbean
16. Any other Black/African/Caribbean background

Other ethnic group
17. Arab
18. Any other ethnic group
