# Supplementary material for: Understanding symptom clusters, diagnosis and healthcare experiences in myalgic encephalomyelitis/chronic fatigue syndrome and long COVID: a cross-sectional survey in the UK
Source: BMJ Open. 2025 Apr 2;15(4):e094658. doi: 10.1136/bmjopen-2024-094658 (PMC11966950; doi:10.1136/bmjopen-2024-094658)
Supplement: online supplemental file 2 [file bmjopen-15-4-s002.docx]

# Appendix

Supplementary Table 1 - Demographic data from all respondents who have, or have a family member who has, a diagnosis or symptoms of ME/CFS/Long Covid.

|  | Under 18 | 18 - 29 | 30 - 39 | 40 - 49 | 50 - 59 | 60 - 69 | 70 - 79 | 80 or older | Didn’t say |
| --- | --- | --- | --- | --- | --- | --- | --- | --- | --- |
| Female | 80 | 469 | 827 | 1337 | 1719 | 1118 | 439 | 49 | 15 |
| Male | 27 | 59 | 107 | 198 | 295 | 249 | 126 | 14 | 2 |
| Non-Binary/ Self-described | 4 | 43 | 42 | 23 | 14 | 3 | 0 | 0 | 0 |

Supplementary Table 2: Age and gender of people with Long Covid and ME

|  | | Under 18 | 18 - 29 | 30 - 39 | 40 - 49 | 50 - 59 | 60 - 69 | 70 - 79 | 80 or older | Didn’t say |
| --- | --- | --- | --- | --- | --- | --- | --- | --- | --- | --- |
| I have a close family member or friend that has been diagnosed with Long Covid | Female | 9 | 1 | 4 | 6 | 4 | 5 | 1 | 0 | 0 |
|  | Male | 5 | 1 | 0 | 2 | 2 | 2 | 2 | 1 | 0 |
|  | Non-binary | 0 | 0 | 0 | 0 | 0 | 0 | 0 | 0 | 0 |
| I have a close family member or friend that has been diagnosed with ME/CFS | Female | 31 | 32 | 15 | 13 | 32 | 20 | 7 | 3 | 3 |
|  | Male | 13 | 16 | 11 | 10 | 3 | 6 | 1 | 0 | 0 |
|  | Non-binary | 2 | 3 | 0 | 0 | 0 | 0 | 0 | 0 | 0 |
| I have a close family member or friend with symptoms of Long Covid but no diagnosis | Female | 0 | 0 | 0 | 2 | 1 | 1 | 2 | 0 | 0 |
|  | Male | 0 | 0 | 0 | 0 | 1 | 2 | 0 | 0 | 0 |
|  | Non-binary | 0 | 0 | 0 | 0 | 0 | 0 | 0 | 0 | 0 |
| I have a close family member or friend with symptoms of ME/CFS but no diagnosis | Female | 2 | 0 | 0 | 1 | 4 | 4 | 0 | 1 | 0 |
|  | Male | 0 | 1 | 0 | 0 | 0 | 1 | 1 | 0 | 0 |
|  | Non-binary | 0 | 0 | 0 | 0 | 0 | 0 | 0 | 0 | 0 |
| I have symptoms and a diagnosis of Long Covid | Female | 7 | 27 | 80 | 150 | 179 | 74 | 17 | 3 | 0 |
|  | Male | 2 | 3 | 14 | 29 | 37 | 23 | 9 | 0 | 0 |
|  | Non-binary | 0 | 2 | 2 | 4 | 2 | 0 | 0 | 0 | 0 |
| I have symptoms and a diagnosis of ME/CFS | Female | 27 | 378 | 685 | 1109 | 1419 | 974 | 391 | 41 | 12 |
|  | Male | 6 | 35 | 76 | 145 | 233 | 203 | 104 | 11 | 2 |
|  | Non-binary | 2 | 32 | 36 | 19 | 12 | 3 | 0 | 0 | 0 |
| I think I have Long Covid symptoms but have not been diagnosed | Female | 1 | 3 | 7 | 11 | 16 | 7 | 12 | 0 | 0 |
|  | Male | 1 | 0 | 1 | 1 | 3 | 6 | 3 | 2 | 0 |
|  | Non-binary | 0 | 0 | 1 | 0 | 0 | 0 | 0 | 0 | 0 |
| I think I have ME/CFS symptoms but have not been diagnosed | Female | 3 | 28 | 36 | 45 | 64 | 33 | 9 | 1 | 0 |
|  | Male | 0 | 3 | 5 | 11 | 16 | 6 | 6 | 0 | 0 |
|  | Non-binary | 0 | 6 | 3 | 0 | 0 | 0 | 0 | 0 | 0 |

Supplementary Table 3: Ethnicity

| **Ethnicity** | **Number** |
| --- | --- |
| **English/Welsh/Scottish/Northern Irish/British** | 6574 |
| **Any other White background** | 306 |
| **Irish** | 89 |
| **Would rather not say** | 78 |
| **Any other Mixed/Multiple ethnic background** | 40 |
| **White and Asian** | 36 |
| **White and Black Caribbean** | 34 |
| **Any other ethnic group** | 33 |
| **Indian** | 31 |
| **(blank)** | 24 |
| **Caribbean** | 9 |
| **Pakistani** | 8 |
| **Any other Asian background** | 8 |
| **Any other Black/African/Caribbean background** | 7 |
| **African** | 7 |
| **White and Black African** | 6 |
| **Chinese** | 6 |
| **Arab** | 3 |
| **Bangladeshi** | 3 |
| **Gypsy or Irish Traveller** | 1 |
| **Grand Total** | **7303** |

Supplementary Table 4: Time of Diagnosis

| **Time of diagnosis for ME/CFS** | **Numbers** | **Percentage** |
| --- | --- | --- |
| Less than 3 months. | 388 | 5.15 |
| 7 – 12 months. | 1419 | 18.84 |
| 1 – 2 years. | 1664 | 22.1 |
| 3 – 4 years. | 883 | 11.72 |
| 3 – 6 months. | 1240 | 16.47 |
| 5 – 6 years. | 461 | 6.12 |
| 7 – 8 years. | 249 | 3.31 |
| 9 – 10 years. | 201 | 2.67 |
| More than 10 years. | 975 | 12.95 |
| Prefer not to say | 51 | 0.68 |
| **Grand Total** | **7531** | **100** |

Supplementary Table 5 - Responses to the question 'What is your overall experience of the NHS in regard to having Long Covid, and/or ME/CFS?'

|  |  | Very Poor | Poor | Mixed | Good | Excellent | Don't know |
| --- | --- | --- | --- | --- | --- | --- | --- |
| Diagnosed Long COVID | Count | 158 | 120 | 258 | 84 | 19 | 8 |
|  | % | 22.13 | 16.81 | 36.13 | 11.76 | 2.66 | 1.12 |
| Diagnosed ME/CFS | Count | 2157 | 1175 | 1631 | 338 | 89 | 34 |
|  | % | 33.76 | 18.39 | 25.52 | 5.29 | 1.39 | 0.53 |

Supplementary Table 6 - Responses to the question 'When did a Specialist last see you?'

| **Condition** | **Timeframe** | **Count** | **Percentage (%)** |
| --- | --- | --- | --- |
| **Long COVID** | In the last month | 84 | 23.66 |
|  | 2-6 months ago | 133 | 37.46 |
|  | 7-11 months ago | 46 | 12.96 |
|  | 1-2 years ago | 60 | 16.90 |
|  | 3-4 years ago | 5 | 1.41 |
|  | 5 years ago or longer | 0 | 0.00 |
|  | I haven't been seen | 24 | 6.76 |
|  | Don't know | 3 | 0.85 |

Supplementary Table 7 - Responses to the question 'How do you manage your symptoms?' Count and percentages of all respondents within the group.

| Symptom management method | Count | Long COVID | | | ME/CFS | | | |
| --- | --- | --- | --- | --- | --- | --- | --- | --- |
|  |  | Diagnosed | Undiagnosed | Total | | Diagnosed | Undiagnosed | Total |
| Cognitive behavioural therapy (to help me cope) | Count | 93 | 5 | 98 | | 855 | 29 | 884 |
|  | % | 13.7 | 6.5 | 12.9 | | 14.0 | 10.3 | 13.8 |
| Counselling (to help me cope) | Count | 171 | 16 | 187 | | 1263 | 39 | 1302 |
|  | % | 25.1 | 20.8 | 24.7 | | 20.7 | 13.9 | 20.4 |
| Occupational therapy | Count | 117 | 2 | 119 | | 512 | 10 | 522 |
|  | % | 17.2 | 2.6 | 15.7 | | 8.4 | 3.6 | 8.2 |
| Graded Exercise Therapy | Count | 23 | 2 | 25 | | 173 | 4 | 177 |
|  | % | 3.4 | 2.6 | 3.3 | | 2.8 | 1.4 | 2.8 |
| Pulmonary Rehabilitation | Count | 29 | 3 | 32 | | 21 | 3 | 24 |
|  | % | 4.3 | 3.9 | 4.2 | | 0.3 | 1.1 | 0.4 |
| Physiotherapy | Count | 104 | 5 | 109 | | 564 | 23 | 587 |
|  | % | 15.3 | 6.5 | 14.4 | | 9.2 | 8.2 | 9.2 |
| I received medication from a GP (prescription) | Count | 316 | 27 | 343 | | 2491 | 63 | 2554 |
|  | % | 46.4 | 35.1 | 45.3 | | 40.7 | 22.4 | 39.9 |
| I’ve been self-treating with over-the-counter medications (from a pharmacy) | Count | 183 | 18 | 201 | | 1088 | 63 | 1151 |
|  | % | 26.9 | 23.4 | 26.5 | | 17.8 | 22.4 | 18.0 |
| I’ve been taking vitamins or supplements | Count | 434 | 43 | 477 | | 3740 | 162 | 3902 |
|  | % | 63.7 | 55.8 | 62.9 | | 61.2 | 57.7 | 61.0 |
| I self-manage without the use of medications | Count | 116 | 19 | 135 | | 1379 | 64 | 1443 |
|  | % | 17.0 | 24.7 | 17.8 | | 22.5 | 22.8 | 22.6 |
| I’ve made changes to my diet | Count | 312 | 27 | 339 | | 2757 | 120 | 2877 |
|  | % | 45.8 | 35.1 | 44.7 | | 45.1 | 42.7 | 45.0 |
| I try to balance activities with rest | Count | 465 | 58 | 523 | | 4382 | 184 | 4566 |
|  | % | 68.3 | 75.3 | 69.0 | | 71.6 | 65.5 | 71.4 |
| I rest and sleep a lot more than before | Count | 550 | 63 | 613 | | 4678 | 221 | 4899 |
|  | % | 80.8 | 81.8 | 80.9 | | 76.5 | 78.6 | 76.6 |
| I’ve had to reduce all activities | Count | 528 | 57 | 585 | | 4908 | 227 | 5135 |
|  | % | 77.5 | 74.0 | 77.2 | | 80.2 | 80.8 | 80.3 |
| I’ve had to reorganise my life and take things a lot easier | Count | 567 | 55 | 622 | | 4939 | 223 | 5162 |
|  | % | 83.3 | 71.4 | 82.1 | | 80.8 | 79.4 | 80.7 |
| Self-management approaches (energy management, pacing etc.) | Count | 433 | 39 | 472 | | 3958 | 139 | 4097 |
|  | % | 63.6 | 50.6 | 62.3 | | 64.7 | 49.5 | 64.0 |
| Alternative or complementary approaches (massage, reiki, homeopathy etc.) | Count | 174 | 12 | 186 | | 1595 | 55 | 1650 |
|  | % | 25.6 | 15.6 | 24.5 | | 26.1 | 19.6 | 25.8 |
| I cope as best I can on my own | Count | 346 | 53 | 399 | | 3342 | 153 | 3495 |
|  | % | 50.8 | 68.8 | 52.6 | | 54.6 | 54.4 | 54.6 |
| I’ve been too sick to be able to try anything | Count | 65 | 5 | 70 | | 787 | 23 | 810 |
|  | % | 9.5 | 6.5 | 9.2 | | 12.9 | 8.2 | 12.7 |
| None of the above | Count | 2 | 0 | 2 | | 7 | 1 | 8 |
|  | % | 0.3 | 0.0 | 0.3 | | 0.1 | 0.4 | 0.1 |
| Other(please specify) | Count | 114 | 3 | 117 | | 935 | 26 | 961 |
|  | % | 16.7 | 3.9 | 15.4 | | 15.3 | 9.3 | 15.0 |
| Prefer not to say | Count | 0 | 0 | 0 | | 9 | 0 | 9 |
|  | % | 0.0 | 0.0 | 0.0 | | 0.1 | 0.0 | 0.1 |
|  | Count | 681 | 77 | 758 | | 6116 | 281 | 6397 |
