# Supplementary material for: Understanding symptom clusters, diagnosis and healthcare experiences in myalgic encephalomyelitis/chronic fatigue syndrome and long COVID: a cross-sectional survey in the UK
Source: BMJ Open. 2025 Apr 2;15(4):e094658. doi: 10.1136/bmjopen-2024-094658 (PMC11966950; doi:10.1136/bmjopen-2024-094658)
Supplement: Supplementary file 3 [file bmjopen-2024-094658.reviewer_comments.pdf]

PEER REVIEW HISTORY

BMJ Open publishes all reviews undertaken for accepted manuscripts. Reviewers are asked to complete a checklist review form and are provided with free text boxes to elaborate on their assessment. These free text comments are reproduced below.

ARTICLE DETAILS

Title (Provisional)

Understanding Symptom Clusters, Diagnosis, and Healthcare Experiences in Myalgic Encephalomyelitis/Chronic Fatigue Syndrome and Long COVID: A Cross-Sectional Survey in the UK

Authors

Mansoubi, Maedeh; Richards, Thomas; Ainsworth-Wells, Martine; Fleming, Russell; Leveridge, Phaedra; Shepherd, Charles; Dawes, Helen

---

VERSION 1 - REVIEW

**Reviewer**	**1**
**Name**	**Berkis, Uldis**
**Affiliation**	**Development and Project Department, Riga Stradins University, Riga, Latvia, Project Department**
**Date**	**04-Nov-2024**
**COI**	**None**

Inclusion/stratification of respondents could clarify the situation when post-COVID fatigue is leading to ICD-10 93.31 diagnosis, i.e. a patient is consequentially both diagnosed/perceived to have ME/CFA and long-COVID at the same time. Of interest of course if ICD-10 U09.9 was used for long-COVID. In the case of surveys, this is a complex issue for respondents to correctly drive the answers.

Have all patients with a diagnosis been diagnosed according to NICE guidelines? They require persistence of symptoms of 3 months, whereas Canada consensus criteria and Fukuda - 6 months, ICC no definite period of time. Various diagnostic approaches will cause differences in time to diagnosis.

Did secondary data refer to the use of aggregate data analysis methods in a secure environment or it is using the survey data for further purposes as when primarily collected?

**Reviewer**	**2**
**Name**	**Barnden, Leighton**

**Affiliation**	**Griffith University, NCNED**
**Date**	**02-Dec-2024**
**COI**	**None**

Please provide access to questionnaire.

p5 line 25: 5) To determine the impact of the NICE ...

p6 line 6: ... confirm whether they were completing for themself or or for a close ...

p6 line 51,52: To -> to

p13 line 40: recency -> recent onset

p13 line 56: evidenced -> evidence based

Figure captions: replace [Field-Specialist] etc with actual field. eg 'GP or consultant'

Supplementary Table captions: replace [Field-Resp Type] etc with readable text.

VERSION 1 - AUTHOR RESPONSE

Reviewer: 1
Dr. Uldis Berkis, Development and Project Department, Riga Stradins University, Riga, Latvia

Comments to the Author:
Inclusion/stratification of respondents could clarify the situation when post-COVID fatigue is leading to ICD-10 93.31 diagnosis, i.e. a patient is consequentially both diagnosed/perceived to have ME/CFA and long-COVID at the same time. Of interest of course if ICD-10 U09.9 was used for long-COVID. In the case of surveys, this is a complex issue for respondents to correctly drive the answers.

Thank you for this insightful comment highlighting the potential overlap between ME/CFS and Long COVID diagnoses, as well as the challenges associated with respondent interpretation of diagnostic categories. We agree that this is a complex issue, particularly for surveys relying on self-reported data. In response, we have revised the manuscript to address this point more clearly:

We have added the following explanation to the Methods section to outline how these diagnostic overlaps were handled: "Respondents were asked to self-identify whether they or a close contact had been diagnosed with ME/CFS or Long COVID. In cases where participants reported overlapping diagnoses (e.g., ME/CFS and post-COVID fatigue leading to an ICD-10 G93.31 diagnosis), they were included in both ME/CFS and Long COVID analyses if responses satisfied the respective diagnostic criteria outlined in the survey."

We have also included a discussion of the limitations of self-reported diagnoses and ICD-10 coding: "The distinction between ME/CFS (ICD-10 G93.31) and Long COVID (ICD-10 U09.9) is complex and may not always be clearly understood by patients or clinicians. This limitation is particularly relevant in cases of post-COVID fatigue where diagnostic ambiguity may arise. While our survey allowed participants to select multiple diagnoses, self-reporting may not capture nuances in clinical criteria, and future studies could benefit from incorporating clinician-verified diagnoses or more specific diagnostic questions to address this overlap."

To address stratification, we have added text to the Results section highlighting the number of respondents who reported dual diagnoses: "Of the participants with ME/CFS or Long COVID, a subset (n = 791) reported both conditions. These cases were analysed separately to explore potential differences in symptom clustering, healthcare experiences, and self-management strategies”

We hope these revisions adequately clarify how respondents with overlapping diagnoses were included in the study and acknowledge the inherent complexities in interpreting ICD-10 coding in the context of self-reported survey data.

Have all patients with a diagnosis been diagnosed according to NICE guidelines? They require persistence of symptoms of 3 months, whereas Canada consensus criteria and Fukuda - 6 months, ICC no definite period of time. Various diagnostic approaches will cause differences in time to diagnosis.

Thank you for highlighting the potential variability in diagnostic approaches and its implications for our study. We acknowledge the diversity of diagnostic criteria for ME/CFS (e.g., NICE guidelines, Canada consensus criteria, Fukuda criteria, ICC) and the challenges this presents in understanding time to diagnosis. Below is our response and the updates made to the manuscript:

Clarification of Diagnostic Criteria in the Questionnaire: As noted in the reviewer’s comment, for respondents without a current diagnosis, the survey included a question asking them to confirm the presence of the four core symptoms specified in the NICE guidelines and their persistence for at least three months. However, there was no specific follow-up to confirm that a current diagnosis adhered to NICE guidelines. This has been clarified in the Methods section with the following addition: "For respondents without a formal diagnosis, the survey asked them to confirm the presence of four key symptoms (fatigue, post-exertional malaise, cognitive dysfunction, and unrefreshing sleep) and their persistence for at least three months, as outlined in the NICE guidelines. For those reporting a current diagnosis, no specific question was included to confirm adherence to NICE or other diagnostic criteria."

Did secondary data refer to the use of aggregate data analysis methods in a secure

environment or it is using the survey data for further purposes as when primarily collected?

Thank you for your query regarding the use of secondary data in this study. We confirm that the secondary data analysis was conducted in accordance with ethical guidelines and the terms under which the data were originally collected. Specifically:

Data Usage Context: The secondary data used in this study refers to the anonymised survey data collected for the primary purpose of understanding the experiences and perspectives of individuals with ME/CFS and Long COVID. This study represents a secondary analysis of that dataset, focusing on additional research questions aligned with the original purpose of the data collection.

Secure Environment: The anonymised survey data were securely stored and analysed in accordance with the University of Exeter's data protection policies. Access to the data was restricted to authorised researchers involved in this study, and all analyses were conducted within a secure environment to ensure compliance with data security and confidentiality requirements.

Clarification Added to Methods Section: To clarify this in the manuscript, we have added the following text to the Methods section: "This study involved secondary analysis of anonymised survey data collected for the primary purpose of exploring the experiences of individuals with ME/CFS and Long COVID. The data were analysed in a secure environment in accordance with the University of Exeter's data protection policies. No new data were collected, and the secondary analysis was conducted within the scope of the original data collection purpose, as outlined to participants at the time of the survey."

We hope this explanation addresses your concern and provides clarity on the ethical and methodological aspects of secondary data usage in this study.

Reviewer: 2
Dr. Leighton Barnden, Griffith University, Griffith University

Comments to the Author:
Please provide access to questionnaire.

Thank you very much for highlighting this issue. We provided the questionnaire as an Appendix.

p5 line 25: 5) To determine the impact of the NICE ...

Thank you very much. We edited the sentence accordingly.

p6 line 6: ... confirm whether they were completing for themself or or for a close ...

Thank you very much. We edited the sentence accordingly.
p6 line 51,52: To -> to

Thank you very much. We edited the sentence accordingly.
p13 line 40: recency -> recent onset

Thank you very much. We edited the sentence accordingly.
p13 line 56: evidenced -> evidence based

Thank you very much. We edited the sentence accordingly.
Figure captions: replace [Field-Specialist] etc with actual field. eg 'GP or consultant'

Thank you very much. We edited the figures captions accordingly.
Supplementary Table captions: replace [Field-Resp Type] etc with readable text.

Thank you very much. We edited the captions accordingly.
